# Olyset Duo® (a Pyriproxyfen and Permethrin Mixture Net): An Experimental Hut Trial against Pyrethroid Resistant *Anopheles gambiae* and *Culex quinquefasciatus* in Southern Benin

**DOI:** 10.1371/journal.pone.0093603

**Published:** 2014-04-03

**Authors:** Corine Ngufor, Raphael N’Guessan, Josias Fagbohoun, Abibatou Odjo, David Malone, Martin Akogbeto, Mark Rowland

**Affiliations:** 1 London School of Hygiene and Tropical Medicine (LSHTM), London, United Kingdom; 2 Centre de Recherches Entomologiques de Cotonou (CREC), Cotonou, Benin; 3 Pan African Malaria Vector Research Consortium (PAMVERC), London, United Kingdom; 4 Innovative Vector Control Consortium (IVCC), Liverpool, United Kingdom; Institut Pasteur, France

## Abstract

**Background:**

Alternative compounds which can complement pyrethroids on long-lasting insecticidal nets (LN) in the control of pyrethroid resistant malaria vectors are urgently needed. Pyriproxyfen (PPF), an insect growth regulator, reduces the fecundity and fertility of adult female mosquitoes. LNs containing a mixture of pyriproxyfen and pyrethroid could provide personal protection through the pyrethroid component and reduce vector abundance in the next generation through the sterilizing effect of pyriproxyfen.

**Method:**

The efficacy of Olyset Duo, a newly developed mixture LN containing pyriproxyfen and permethrin, was evaluated in experimental huts in southern Benin against pyrethroid resistant *Anopheles gambiae* and *Culex quinquefasciatus*. Comparison was made with Olyset Net® (permethrin alone) and a LN with pyriproxyfen alone (PPF LN). Laboratory tunnel tests were performed to substantiate the findings in the experimental huts.

**Results:**

Overall mortality of wild pyrethroid resistant *An. gambiae* s.s. was significantly higher with Olyset Duo than with Olyset Net (50% vs. 27%, P = 0.01). Olyset DUO was more protective than Olyset Net (71% vs. 3%, P<0.001). The oviposition rate of surviving blood-fed *An. gambiae* from the control hut was 37% whereas *none* of those from Olyset Duo and PPF LN huts laid eggs. The tunnel test results were consistent with the experimental hut results. Olyset Duo was more protective than Olyset Net in the huts against wild pyrethroid resistant *Cx. quinquefasciatus* although mortality rates of this species did not differ significantly between Olyset Net and Olyset Duo. There was no sterilizing effect on surviving blood-fed *Cx. quinquefasciatus* with the PPF-treated nets.

**Conclusion:**

Olyset Duo was superior to Olyset Net in terms of personal protection and killing of pyrethroid resistant *An. gambiae*, and sterilized surviving blood-fed mosquitoes. Mixing pyrethroid and pyriproxyfen on a LN shows potential for malaria control and management of pyrethroid resistant vectors by preventing further selection of pyrethroid resistant phenotypes.

## Background

Malaria vector control relies primarily on two interventions: long lasting insecticidal nets (LNs) and indoor residual spraying (IRS). Both interventions have contributed significantly to the recent reductions in malaria morbidity and mortality observed across sub-Saharan Africa [1]. While several classes of insecticide can be used for IRS, the pyrethroids are currently the only class of insecticide recommended by the World Health Organisation (WHO) for treating LNs owing to their safety, excito-repellency and rapid knock down effect. Pyrethroid resistance has become widespread in malaria vectors in several malaria endemic parts of the world [2]. Recent reports across Africa have shown that pyrethroid resistance threatens to undermine the effectiveness of LNs and without prompt action, the benefits so far achieved in the control of malaria could be reversed [3], [4].

The prospects for identifying alternative insecticides to pyrethroids for treating mosquito bed-nets are limited [3]. Most alternatives tested on mosquito nets are too toxic to mammals and lack the excito-repellent property inherent in pyrethroids; hence they provide little or no direct personal protection to users [5]–[8]. However, without LNs as a vehicle for insecticide, it is unlikely that the goal of universal coverage with personal protection can be achieved or sustained in most malaria endemic communities [3]. Strategies to preserve the efficacy of LNs in the era of pyrethroid resistance are therefore paramount. Mosquito nets can be treated with a combination of pyrethroid and non pyrethroid insecticide to which vectors are susceptible. This approach provides an opportunity to preserve the protectiveness of the net through the excito-repellent properties of the pyrethroid while enhancing toxicity through the non-pyrethroid alternative [9]. Use of mixtures on nets has the potential to manage insecticide resistance if insects resistant to one insecticide are susceptible to and killed by the other [4], [10], [11].

Pyriproxyfen is an insect juvenile hormone mimic, recommended for larval control by WHO [12], [13]. It is safe to humans and shows no cross resistance to other classes of insecticides used for vector control [14]. The primary use of pyriproxyfen is as an insect growth regulator to inhibit adult emergence hence its use for mosquito control has been limited to larval stages [12], [15], [16]. However, pyriproxyfen has also been reported to inhibit oogenesis and sterilize adult mosquito vectors [17]. Studies with adult *Aedes aegypti* have demonstrated reduced fecundity in females which have tarsal contact with pyriproxyfen treated substrates [18], [19]. Earlier studies on Anophelines demonstrated reduced fertility in the eggs oviposited by *Anopheles stephensi* females exposed to pyriproxyfen treated netting [20]. More recent studies have shown complete sterilization of *An. gambiae* females exposed to pyriproxyfen treated netting [17] and *An. arabiensis* females exposed one day after feeding to pyriproxyfen in CDC bottle bioassays [21].

Mixing pyriproxyfen with pyrethroids on mosquito nets could provide a combination of personal protection through the pyrethroid component and mass population effect on the next generation of vectors through the sterilizing effect of the pyriproxyfen component on parental females. Such a mixture LN is expected to be effective against a wide range of mosquito species including those with multiple mechanisms of resistance to current insecticides. It could also slow the spread of pyrethroid resistance genes if deployed in areas where pyrethroid resistance is still rare. In the current study, we evaluated the efficacy of Olyset Duo (Sumitomo Chemical Company Ltd); a newly developed pyriproxyfen and permethrin incorporated polyethylene LN in experimental huts against wild, free flying pyrethroid resistant *An. gambiae* and *Cx. quinquefasciatus* in Southern Benin where both mosquito species are highly resistant to pyrethroids. Comparison was made to a WHOPES-recommended LN treated with permethrin alone (Olyset Net; Sumitomo Chemical Company Ltd) and a LN treated with pyriproxyfen alone, which was formulated to the same technical specifications as Olyset Duo. Studies with resistant strains were also carried out using laboratory tunnel tests to corroborate the findings in the experimental huts.

## Materials and Methods

### Study Site and Experimental Huts

The study was carried out at the CREC experimental hut station in Akron, a village on the outskirts of Porto Novo, Benin. The site supports breeding of *An. gambiae* M form that are pyrethroid-resistant due to high frequency of *kdr* (>90%) and increased activity of cytochrome P450s [22]. The nuisance mosquito *Cx. quinquefasciatus* is present year round and shows resistance to pyrethroids, carbamates and organophosphates [22]. Four experimental huts of the West African design as recommended by WHO were used for the study. The huts are built on concrete plinths surrounded by water-filled moats to prevent entry of scavenging ants. Mosquitoes exiting the huts are captured by veranda traps. The huts are made of brick plastered with cement on the inside, with a corrugated iron roof and have a ceiling of palm thatch and four window slits (1 cm gap) on their walls through which mosquitoes enter.

### Treatments and Trial Procedure

The following four treatments were tested in the experimental huts.

1. Untreated control mosquito net (polyethylene net),
2. Pyriproxyfen (PPF) LN (Sumitomo Chemical Co. Ltd., Tokyo, Japan),
3. Olyset Net® (Sumitomo Chemical Co. Ltd., Tokyo, Japan) – a WHOPES-recommended standard permethrin incorporated LN,
4. Olyset Duo® (Sumitomo Chemical Co. Ltd., Tokyo, Japan) – a newly developed 1% w/w pyriproxyfen and 2% w/w permethrin incorporated LN.

Olyset DUO and Olyset Net have the same concentration of permethrin. Olyset DUO however has a faster permethrin bleed rate (rate of release from the net fibres to the surface) than Olyset Net. Preliminary laboratory studies revealed a shorter regeneration time of permethrin in Olyset DUO (3days) than Olyset Net (7days) confirming the faster rate. PPF LN does not contain permethrin but has a similar pyriproxyfen bleed rate as Olyset DUO.

To simulate wear and tear, the bed nets were intentionally holed with six 16 cm^2^ holes (two holes on each side and one on each end) according to WHOPES guidelines [23]. Treatments were allocated to the experimental huts on a weekly basis following a Latin square design to adjust for any variation in site attractiveness of the huts. Four adult human volunteers were offered chemoprophylaxis and slept in the huts from 20∶00 to 05∶00 each night of the study; they were rotated between huts on successive nights to adjust for any variation in individual attractiveness to mosquitoes.

### Outcome Measures

Mosquitoes were collected each morning at 05∶00 from under bed nets, floors, walls, ceilings and verandas using aspirators and torches. The collections were transported to the laboratory where the mosquitoes were morphologically identified to genus/species using taxonomic keys and samples of *An gambiae* were confirmed as M form [24]. They were then scored as blood fed or unfed and live or dead. Live mosquitoes were held in netted plastic cups and supplied with 10% glucose solution and delayed mortality was recorded after 24 h. Male mosquitoes were not scored.

Because pyriproxyfen acts by sterilizing the adult female mosquito, the impact of the treatments on the reproduction of surviving blood-fed mosquitoes was investigated by detecting whether there was a reduction in the fecundity (number of eggs per female) and fertility (proportion of laid eggs hatching) of these mosquitoes compared to the control. After scoring for mortality (24 h post-collection from the experimental huts), the live blood-fed mosquitoes of each treatment were kept in separate cages and provided access to a second blood meal. Once gravid (within 2–3 days), individual mosquitoes were chambered separately in their own netted plastic cups containing approximately 50 ml of fresh water. The chambers were monitored daily for eggs and the number of eggs laid by each female mosquito was recorded for up to 9 days. A pinch of larval food was added to any chamber which contained eggs and the numbers of larvae (L2) which hatched were recorded after another 4–6 days.

For each type of LN, the efficacy in experimental huts and the sterilizing effect on mosquitoes which survived the hut treatments were studied using the following outcome measures.

Direct effects on adult females in experimental huts:

1. Deterrence: percentage reduction in the number of mosquitoes caught in treated hut relative to the number caught in the control hut
2. Exiting rates: due to potential irritant effect of treatments expressed as percentage of the mosquitoes collected from the veranda trap
3. Inhibition of blood-feeding: reduction in blood-feeding rate relative to the control. Blood feeding inhibition (%) was calculated as follows:
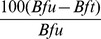

Where *Bfu* is the proportion of blood-fed mosquitoes in the untreated control huts and *Bft* is the proportion of blood-fed mosquitoes in the huts with a specific insecticide treatment.

4. Mortality: percentage of dead mosquitoes in treated hut at the time of collection and after a 24 h holding period corrected for control mortality.
5. The personal protective effect of the treatments which is described by a reduction in the number of blood-fed mosquitoes relative to the control hut. Personal protection (%) was calculated as follows:
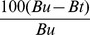

Where *Bu* is the number of blood-fed mosquitoes in the untreated control huts and *Bt* is the number of blood-fed mosquitoes in the huts with insecticide treatments.

6. The overall insecticidal effect of a treatment relative to the number of mosquitoes that would ordinarily enter an untreated control hut. Overall insecticidal effect (%) was estimated by using the following formula:
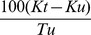

where *Kt* is the number killed in the treated hut, *Ku* is the number dying in the untreated control hut, and *Tu* is the total number collected from the control hut.

Effects on sterility and reproduction of surviving blood-fed females:

1. The proportion of females ovipositing: proportion of blood-fed females which laid eggs.
2. Fecundity: the number of eggs per blood fed female observed.
3. Reproductive rate: the number of larvae per blood fed female observed.
4. Fertility: the hatch rate of eggs laid by females of a given treatment
5. Reduction in fecundity: the percentage reduction in number of eggs per surviving blood fed female observed for a given treatment relative to the control. This was calculated as follows:
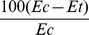

Where *Ec* is the mean number of eggs per surviving blood-fed female observed in the control while *Et* is the mean number of eggs per surviving blood-fed female observed in a given treatment.

6. Reduction in reproductive rate: the percentage reduction in number of larvae per surviving blood fed female observed for a given treatment relative to the control. This was calculated as follows:
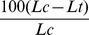

Where *Lc* is the mean number of larvae per surviving blood-fed female observed in the control while *Lt* is the mean number of larvae per surviving blood-fed female observed in a given treatment.

### Tunnel Tests

To gain further insight, laboratory tunnel tests were undertaken on netting samples taken from the hut LNs using the *An. gambiae* VKPER strain which was fixed for the pyrethroid knockdown resistance (*kdr*) gene. The tunnel test allows expression of the behavioural interactions that occur between free-flying mosquitoes and LNs during host seeking. It consists of a square glass cylinder (25 cm high, 25 cm wide, 60 cm in length) divided into two sections by means of a netting frame fitted into a slot across the tunnel [23]. In one of the sections, a guinea pig was housed unconstrained in a small cage, and in the other section 50 unfed female mosquitoes aged 5–8 days were released at dusk and left overnight. The net samples measured 25 cm×25 cm and were deliberately holed with nine 1-cm holes to give opportunity for mosquitoes to penetrate into the animal baited chamber for a blood meal; an untreated net sample served as the control. The tests were performed at 25–27°C and 75–85% RH. The next morning, the numbers of mosquitoes found alive or dead, fed or unfed, in each section were scored. Live mosquitoes were provided with 10% glucose solution and delayed mortality recorded after 24hours. Approximately 100 adult females in 2 replicate tunnel tests were tested on each type of netting. Blood-fed mosquitoes which remained alive after 24 hrs were assessed for sterilizing effects of pyriproxyfen as described above.

### Susceptibility Testing

WHO resistance test kits lined with 0.75% permethrin-treated papers were used to determine the frequency and the strength of resistance to permethrin in *An. gambiae* mosquitoes of the VKPER strain and wild Akron strain relative to the susceptible Kisumu strain. A range of exposure times (1–120 minutes) were tested on batches of 20 unfed *An. gambiae* female 2–5 day old Akron and Kisumu strains. Eighty mosquitoes per exposure period were tested. Deaths were scored 24 h later. Log-time mortality curves were generated using probit analysis and estimates of the time required to kill 50% (LT50) of each strain and the resistance ratios relative to the susceptible laboratory strain (PoloPlus version 1.0).

### Statistical Analysis

The effects of the experimental hut treatments on each of the proportional outcomes (net penetration, blood-feeding, exiting and mortality) were assessed using binomial generalised linear mixed models (GLMMs) with a logit link function, fitted using the ‘lme4’ package for R. A separate model was fitted for each outcome. In addition to the fixed effect of each treatment, each model included random effects to account for the following sources of variation: between the 4 huts; between the 4 sleepers; between the weeks of the trial; and finally an observation-level random effect to account for variation not explained by the other terms in the model (over dispersion). Differences in deterrence, personal protection and mass killing effect between the treatments was analysed using negative binomial regression with adjustment for the abovementioned covariates.

The proportions of surviving blood-fed females from the different treatments that laid eggs was analysed using Chi-square. The proportions of eggs that hatched to larvae was analysed using logistic regression while the numbers of eggs laid and the numbers of larvae per surviving female were analysed using the Kruskal Wallis test. These analyses were performed using STATA version 11.1 Texas USA.

### Ethics Statement

Ethical approval for the study was obtained from the Ethics Review Board of the London School of Hygiene and Tropical Medicine and from the Ministry of Health of Benin. Permission to use the experimental hut station was obtained from ‘Centre de Recherches Entomologique de Cotonou’. Written informed consent was obtained from the volunteers who slept in the experimental huts to attract mosquitoes.

## Results

### Susceptibility Tests

The summary results of the exposure time mortality bioassays with permethrin-treated papers in WHO cylinder kits are shown in Table 1. An accurate LT50 value could not be determined for the laboratory susceptible *An. gambiae* Kisumu strain since mortality rates >90% were achieved within 1 minute of exposure. LT50 values were 6.92 minutes for the *An. gambiae* VKPER strain and 19.48 minutes for wild *An. gambiae* from Akron. The results thus showed that the *An. gambiae* VKPER strain and the wild *An. gambiae* from Akron were at least 6.9 and 19.4 fold more resistant to permethrin than the laboratory susceptible *An. gambiae* Kisumu strain (Table 1). The wild *An. gambiae* from Akron was 2.8 times more resistant to permethrin than the *An. gambiae* VKPER strain.

**Table 1:** Susceptibility of mosquito strains to permethrin-treated papers (0.75%) in WHO cylinder bioassays.

Strains	Slope	LT50$ (minutes)	(95% CI)	LT50 ratio
*An. gambiae* Kisumu	0.68	<1	–	–
*An. gambiae* VKPER	1.58	6.92	4.95–9.39	∼7
*An. gambiae* Akron (wild)*	3.73	19.48	17.05–22.17	∼20

*samples were collected as larvae from breeding sites close to the experimental huts in Akron during the trial,

$ LT50 = time taken for 50% of mosquitoes to be killed.

### Experimental Hut Trial

#### 1. *Anopheles gambiae*

**Figure 1:**
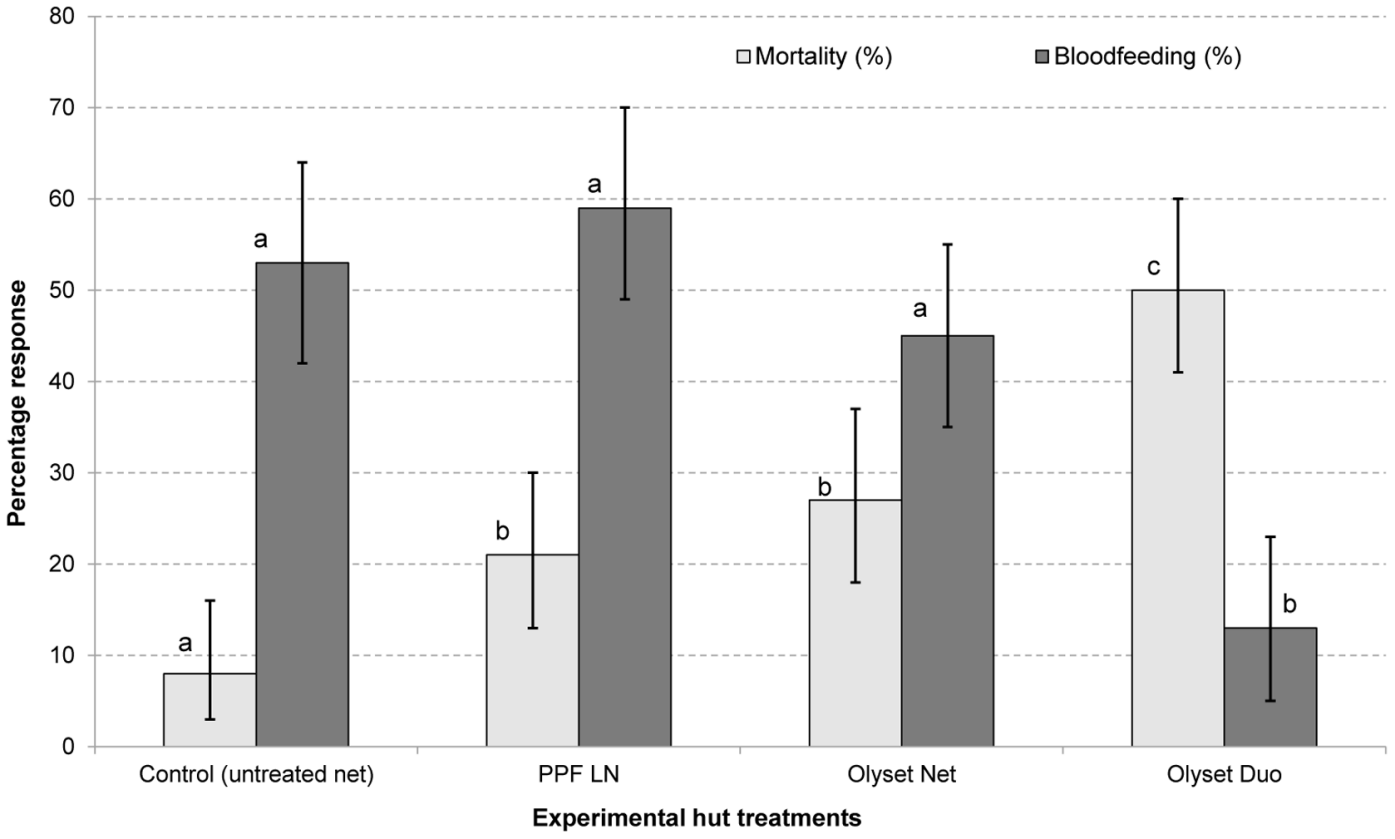
Mortality and bloodfeeding rates of pyrethroid resistant *Anopheles gambiae* in experimental huts. Percentage mortality (lighter shade) and bloodfeeding (darker shade) of pyrethroid resistant *An. gambiae* in experimental huts in Akron. For each response parameter (mortality or bloodfeeding), values for histograms sharing the same letter label are not significantly different (P>0.05). Error bars represent 95% confidence intervals.

Blood feeding and mortality: A total of 303 *An. gambiae* were collected from the experimental huts during the trial. The numbers entering each of the treated huts per night were higher than in the control, hence there was no evidence of a deterrent effect on *An. gambiae* with any of the treatments (Table 2). The proportion exiting from huts with control nets (31%) did not differ significantly from that with PPF LN (29%; P = 0.72) (Table 2). Exiting rates were much higher from huts with Olyset Duo (52%) and Olyset Net (56%) which might be attributed to the excito-repellent property of permethrin in both nets. Percentage blood-fed with the PPF LN (59%) did not differ significantly from the control net (53%, P = 0.44) or Olyset Net (45%, P = 0.07) (Figure 1). The lowest blood-feeding rate was achieved with Olyset Duo (13%). Olyset Duo also provided significantly higher levels personal protection (71%) than Olyset Net (3%, P<0.001) and PPF LN (0%, P<0.001) (Table 3). Lower proportions of mosquitoes were collected from inside the permethrin treated nets (Olyset Net: 11% and Olyset Duo: 4%) than from the PPF LN (35%, P<0.001) or control nets (39%, P<0.001) (Table 3). The proportion collected from inside Olyset Net (11%) did not differ significantly from that from Olyset Duo (4%, P = 0.07). Mortality with PPF LN (21%) was higher than the control net (8%, P = 0.03) but did not differ significantly from Olyset Net (27%, P = 0.24) (Figure 1). Much higher mortality rates were achieved with Olyset Duo than with Olyset Net (50% vs 27%; P = 0.01) and PPF LN (50% vs 21% P<0.001). Olyset Duo induced a higher overall killing effect on *An. gambiae* than did Olyset Net (48% vs 27%, P<0.05) (Table 4).

**Table 2:** Entry and exiting rates of wild mosquitoes in experimental huts during the trial.

	Untreated net	PPF LN	Olyset Net	Olyset Duo
***Anopheles gambiae***				
Total females caught	64	91	76	72
Average catch per night	1.1^a^	1.6^a^	1.3^a^	1.3^a^
% Deterrence	–	0	0	0
Total females exiting	20	26	40	40
% Exiting	31^a^	29^a^	53^b^	56^b^
***Culex quinquefasciatus***				
Total females caught	1331	1456	1597	1505
Average catch per night	23.4^a^	25.5^a^	28.0^a^	26.4^a^
% Deterrence	–	0	0	0
Total females exiting	375	488	908	943
% Exiting	29^a^	32^b^	59^c^	66^d^

a,b,c,d Numbers in the same row sharing a letter superscript do not differ significantly (P>0.05).

**Table 3:** Blood-feeding inhibition and personal protection rates in the experimental huts.

	Untreated net	PPF LN	Olyset Net	Olyset Duo
***Anopheles gambiae***				
Total blood fed	35	54	34	10
% Blood fed	53^a^	59^a^	45^a^	13^b^
% Blood feeding inhibition	–	0^a^	15^b^	75^c^
% Personal Protection	–	0^a^	3^a^	71^b^
% Inside net	39^a^	31^a^	11^b^	4^b^
***Culex quinquefasciatus***				
Total blood fed	510	612	240	32
% Blood fed	36^a^	43^a^	11^b^	2^c^
% Blood feeding inhibition	–	0^a^	69^b^	94^c^
% Personal Protection	–	0^a^	53^b^	92^c^
% Inside net	39^a^	35^b^	9^c^	4^d^

a,b,c,d Numbers in the same row sharing a letter superscript do not differ significantly (P>0.05).

**Table 4:** Overall killing effect in the experimental huts.

	Untreated net	PPF LN	Olyset Net	Olyset Duo
***Anopheles gambiae***				
Total females dead	4	19	21	36
Corrected mortality	0^a^	14^b^	21^b^	46^c^
% Overall killing effect	–	23^a^	27^a^	48^b^
***Culex quinquefasciatus***				
Total females dead	50	152	212	228
Corrected mortality	0^a^	5^b^	9^c^	10^c^
% Overall killing effect	–	8^a^	12^b^	13^b^

a,b,c Numbers in the same row sharing a letter superscript do not differ significantly (P>0.05).

Reproductive effects: The impact of the different LNs on the fecundity and reproductive rate of surviving blood fed female *An. gambiae* from the experimental huts (alive after 24 h) are presented in Table 5. The numbers of blood-fed pyrethroid resistant mosquitoes surviving the hut treatments and the numbers observed for sterilizing effects were both very small. Nevertheless the sterilizing effect of the pyriproxyfen-treated nets on *An. gambiae* was very obvious. The proportions from the control hut which laid eggs was 37% resulting in an average of 37 eggs per female observed with 98% hatching to larvae (Table 5). The numbers of blood-fed mosquitoes from the Olyset Net hut which laid eggs and the number of eggs and larvae per female were higher but not significantly higher than with the control. None of the surviving blood fed females from the huts with PPF LN or Olyset Duo laid eggs. Hence the pyriproxyfen-treated nets (PPF LN and Olyset Duo) completely sterilized the surviving blood-fed mosquitoes resulting in 100% reductions in the fecundity and reproductive rate of these mosquitoes relative to the control (Table 5).

**Table 5:** Fecundity and Fertility of blood-fed *An. gambiae* females alive after 24 h from experimental huts.

	Control	PPF LN	Olyset Net	Olyset Duo
No. of blood fed females observed	27	19	15	8
% of females that oviposited (95% CI)	37(17–57)^a^	0^b^	47(20–74)^a^	0^ b^
Total number of eggs laid	1003	0	850	0
Eggs per female laying eggs (95% CI)	100	–	121	–
Fecundity: eggs per blood fedfemale observed (95% CI)	37(15–58)^a^	0^b^	57(30–74)^a^	0^b^
% reduction in fecundity perfemale observed	–	100	–	100
Total number of larvae	981	0	782	0
Hatch rate %, (95% CI)	98 (97–99)^a^	–	92 (90–94)^b^	–
Larvae per female layingeggs (95% CI)	98	–	112	–
Larvae per female observed(95% CI)	36(14–57)^a^	0^b^	52(39–71)^a^	0^b^
% reduction in reproductive rateper blood fed female observed	–	100	0	100

a,b Values along each row sharing the same letter superscript are not significantly different at the 5% level.

#### 2. *Culex quinquefasciatus*

**Figure 2:**
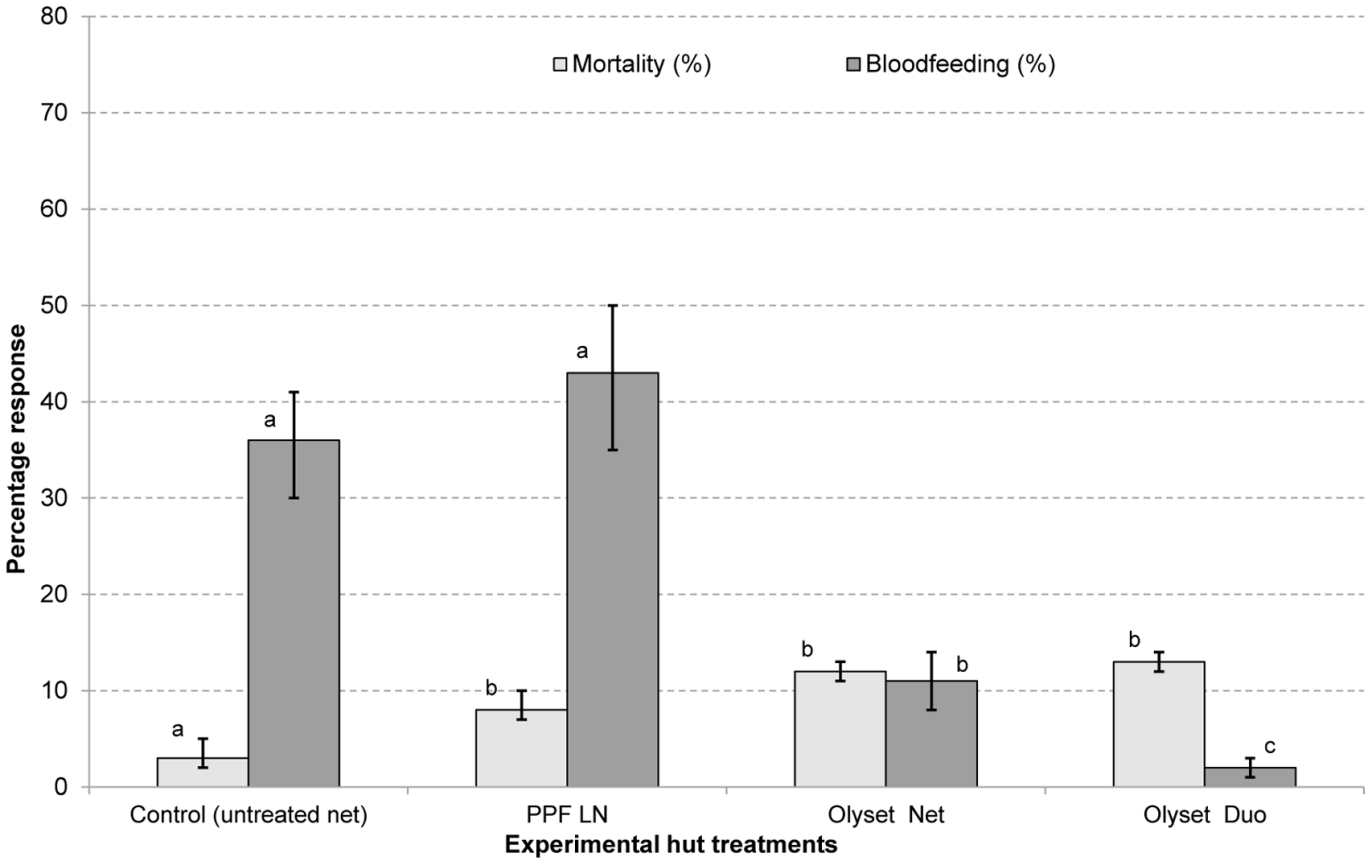
Mortality and bloodfeeding rates of pyrethroid resistant *Culex quinquefasciatus* in experimental huts. Percentage mortality (lighter shade) and bloodfeeding (darker shade) of pyrethroid resistant *Culex quinquefasciatus* in experimental huts in Akron. For each response parameter (mortality or bloodfeeding), values for histograms sharing the same letter label are not significantly different (P>0.05). Error bars represent 95% confidence intervals.

Blood feeding and mortality: A total of 5889 *Cx. quinquefasciatus* were collected from the experimental huts during the trial. There was no evidence of a deterrent effect on this species with any of the treatments (Table 2). The proportions dead and blood-fed are presented in Figure 2. Blood feeding rates with PPF LN (36%) did not differ significantly from the control (43%, P = 0.09). The proportion blood-fed with the permethrin treated nets (Olyset Net = 12% and Olyset Duo = 2%) was significantly lower than with the control or PPF LN (P<0.05). The proportion collected from inside the LN was significantly lower with Olyset Duo (4%) than Olyset Net (9%, P<0.001). Olyset Duo also provided more personal protection (92%) than Olyset Net (53%, P<0.001) and PPF LN (0%, P<0.001) (Table 3). Exiting rates were higher with Olyset Duo (66%) than with Olyset Net (59%, P = 0.001) and PPF LN (32%, P<0.001) (Table 2). Mortality with Olyset Net (12%) was higher than with PPF LN (8%, P = 0.01) and both were significantly higher than the control (3%, P<0.001). However, unlike with *An. gambiae*, mortality of *Cx. quinquefasciatus* with Olyset Duo (13%) did not differ significantly from that with Olyset Net (12%, P = 0.27) (Figure 2 and Table 4). The overall killing effect did not differ between the LNs either (12% vs 13%, P = 0.35).

Reproductive effects: Table 6 presents the effects of the different LN types on the fecundity and fertility of random samples of blood-fed *Cx. quinquefasciatus* mosquitoes which survived the experimental hut treatments (alive after 24 h). The proportion that laid eggs and the number of eggs per female did not differ significantly between any of the treatments and the control (P>0.05). In contrast to *An. gambiae*, there was little or no reduction in fecundity of live blood-fed *Cx. quinquefasciatus* from huts with PPF LN (3%) or Olyset Duo (0%). The hatch rates of eggs laid by mosquitoes from huts with PPF LN (72%) and Olyset Duo (98%) did not differ significantly from the control (85%, P>0.05). There was a small reduction in offspring per live blood-fed female *Cx. quinquefasciatus* observed from the PPF LN (20%). No reduction in fecundity or offspring was detected with samples from the Olyset Duo (0%) (Table 6).

**Table 6:** Fecundity and Fertility of blood-fed *Cx. quinquefasciatus* alive after 24 h from experimental huts.

	Control	PPF LN	Olyset Net	Olyset Duo
No. of blood fed females observed	102	108	83	36
% of females that oviposited (95% CI)	34(22–44)^a^	31(22–40)^a^	30(21–41)^a^	44 (28–62)^a^
Total number of eggs	4287	4398	3239	2159
Eggs per female laying eggs	122	129	130	135
Fecundity: eggs per blood fedfemale observed (95% CI)	42(30–52)^a^	41(29–53)^a^	39(26–52)^a^	58(33–84)^a^
% reduction in fecundity perfemale observed	–	3	7	0
Total number of larvae	3634	3171	2753	2116
Hatch rate (%) (95% CI)	85 (84–86)^a^	72(71–74)^b^	85(84–86)^a^	98(97–99)^c^
Larvae per female layingeggs (95% CI)	104	96	109	132
Larvae per female observed(95% CI)	36 (24–47)^a^	29 (19–40)^a^	35(21–48)^a^	58(32–83)^a^
% reduction in reproductive rateper blood fed female observed	–	20	8	0

a,b,c Values along each row sharing the same letter superscript are not significantly different at the 5% level.

### Tunnel Test

The tunnel test results with the *An. gambiae* VKPER laboratory strain are presented in Table 7. The proportion penetrating the net was 95% with the control and 100% with PPF LN. Net penetration rates were significantly reduced with the two permethrin treated nets and the difference was greater with Olyset Duo (16%) than with Olyset Net (63%, P<0.05). The proportion feeding on the bait showed a pattern consistent with penetration. None of the mosquitoes in the tunnel with Olyset Duo succeeded in feeding (0% blood-fed). Blood feeding inhibition was higher with Olyset Duo (100%) than with Olyset Net (68%) or PPF LN (0%). The trend of blood feeding inhibition was very similar to what was observed in the experimental huts (Table 3). Mortality was 0% in the control tunnel and 3% in the PPF LN tunnel. Mortality increased significantly with the permethrin treatments and as in the hut trial was significantly higher with Olyset Duo (100%) than with Olyset Net (91%, P<0.05) (Figure 1). However, the mortality rates recorded in the tunnel tests were much higher than the rates observed in the experimental huts and this might be attributable to the weaker resistance in the VKPER strain compared to the wild mosquitoes.

**Table 7:** Tunnel test results with *An. gambiae* VKPER.

Net Sample	N	Mortality (%)	Penetration (%)	Blood-fed (%)	Blood feedinginhibition (%)	% Blood-fedand alive (n)
Control	112	0^a^	95^a^	93^a^	–	93^a^ (104)
95% CI		0–5	89–98	86–97		86–97
PPF LN	114	5^a^	100^a^	95^a^	0^a^	91^a^ (104)
95% CI		2–8	96–100	89–98		84–96
Olyset Net	92	91^b^	63^b^	30^b^	68^b^	9^b^ (8)
95% CI		84–96	52–73	21–41		4–16
Olyset Duo	110	100^c^	16^c^	0^c^	100^c^	0^c^ (0)
95% CI		97–100	10–25	0–3		0–3

a,b,c Values along each column sharing the same letter superscript are not significantly different at the 5% level.

The effects on the reproduction of blood-fed mosquitoes which survived the tunnel test treatments are presented in Table 8. Because Olyset Duo tunnel test killed all the mosquitoes it was not possible to assess the sterilizing effect of Olyset Duo on *An. gambiae* VKPER in the tunnel bioassays. The proportion from the control tunnel which laid eggs was 34% with each laying female producing an average 106 eggs. With PPF LN, the proportion which laid eggs was 4% and none of these eggs hatched to larvae. This resulted in a 99% reduction in fecundity and a 100% reduction in reproductive rate with PPF LN relative to the control. The tunnel tests therefore corroborated the experimental hut trials by also showing an improved killing and protective effect with Olyset Duo compared to Olyset Net and the complete sterilization of *An. gambiae* VKPER exposed to PPF LN.

**Table 8:** Fecundity and fertility of *An. gambiae* VKPER alive after exposure to LN samples in tunnel tests.

	Control	PPF LN	Olyset Net
No. of blood fed females observed	104	104	8
% laid (95% CI)	34 (25–44)^a^	4 (1–10)^b^	38 (9–75)^a^
Total number of eggs	3720	24	230
Eggs per female laying eggs	106	6	77
Fecundity: eggs per blood fedfemale observed (95% CI)	32 (20–54)^a^	0.2^b^	29 (2–53)^a^
% reduction in fecundityper female observed	–	99	0
Total number of larvae	1740	0	190
Hatch rate (%) (95% CI)	47 (46–49)^a^	0^b^	83 (77–87)^c^
Larvae per female laying eggs	50	0	95
Larvae per bloodfed femaleobserved (95% CI)	17 (11–30)^a^	0^b^	24 (1–50)^a^
% reduction in reproductiverate per blood fed female observed	–	100	0

a,b,c Values along each row sharing the same letter superscript are not significantly different at the 5% level.

## Discussion

Providing universal coverage of LNs to populations at risk has become a priority for national malaria control programmes in recent years [11]. In areas where vectors are largely susceptible to pyrethroids, LNs are highly effective and the levels of mortality and personal protection achieved in experimental hut trials against such vector populations usually exceed 80% [25], [26]. In the current study, mortality rates and personal protection with the WHOPES-recommended LN (Olyset Net) were very much lower (27% and 3% respectively). This serves to confirm the poor performance of standard LNs reported in several studies in Southern Benin which is due to the presence of multiple mechanisms of pyrethroid resistance in *An. gambiae* in this region [22], [26]–[28]. Olyset Duo demonstrated superior performance to Olyset Net in the experimental huts against this resistant population in terms of higher levels of mortality and personal protection. Although both LNs contain the same concentrations of permethrin, the bleed rate of the insecticide is higher in Olyset Duo than Olyset Net. The surface concentration of permethrin is therefore likely to be higher in Olyset Duo and this may potentially account for the higher mortality rates and personal protection observed with Olyset Duo. Nevertheless, the PPF LN did cause some mortality by itself both in the huts and laboratory studies [20] which may mean there could be an additive effect of the two active ingredients in Olyset Duo. Bioassay studies with the two AIs alone and together in dipped nets are the simplest approach to distinguish between the possibilities of faster bleed rate inducing additional mortality of resistant mosquitoes and interaction between independently acting insecticides.

While it is encouraging that Olyset Duo provided additional mortality of *An. gambiae* and greater personal protection compared to Olyset Net, the main rationale behind incorporating pyriproxyfen was to reduce the size of the first filial generation by reducing the reproductive rate of the parental generation through sterilization. While the number of surviving mosquitoes collected from the Olyset Duo treatment arm was limited, the trial did provide encouraging support for that expectation. The results show that pyrethroid resistant *An. gambiae* that contact the net in the course of feeding and which fail to be killed by a pyrethroid-only LN treatment owing to their resistant status can be sterilized if the LN also contains pyriproxyfen. This would predict that greater reductions in the abundance of pyrethroid resistant malaria vectors would be achieved with community wide use of Olyset Duo than with LNs treated only with pyrethroids. In effect Olyset Duo acts rather like a larvicide – acting to reduce the number of F1 progeny reaching adulthood in the next generation. However, owing to the small numbers of surviving blood-fed mosquitoes collected and observed for reproductive effects - a clear limitation of the study - care should be taken not to over interpret these encouraging results. Proof that better reductions in transmission can be achieved with Olyset DUO than Olyset Net will require a fully-powered, large scale community randomised trial in discrete clusters with their own breeding sites.

By selectively sterilizing surviving pyrethroid-resistant *An. gambiae*, Olyset Duo also shows potential to slow down or prevent further selection of pyrethroid resistance. However, because the benefits of a resistance management approach are less likely to be attained in areas where resistance is well established [10], the nets will need to be deployed in areas where resistance is still rare in order to fully test such a resistance management strategy. In the first instance further hut trials involving mixed susceptible and resistant populations are needed to investigate the potential capacity of Olyset Duo to prevent selection of the pyrethroid resistance.

In contrast to *An. gambiae*, mortality rates of wild pyrethroid resistant *Cx. quinquefasciatu*s in the huts with Olyset Duo did not differ significantly from that with Olyset Net. The pyriproxyfen-treated nets (Olyset Duo and PPF LN) similarly failed to sterilize surviving blood-fed *Cx. quinquefasciatu*s mosquitoes. *Cx. quinquefasciatus* from West Africa are difficult to control with pyrethroids due to resistance involving multiple mechanisms [22], [29]; hence the low mortality rates in this species with either LN was not unexpected. There could be inherent differences in the physiology, behaviour, contact or up-take of pyriproxyfen between *Cx. quinquefasciatus* and *An. gambiae* that might have lessened the chances of blood-fed *Cx. quinquefasciatus* mosquitoes being sterilized by the pyriproxyfen-treated nets. Blood feeding inhibition was significantly higher against *Cx. quinquefasciatus* than *An. gambiae* across all treatments hence the surviving *Cx. quinquefasciatus* mosquitoes may not have contacted the nets long enough to pick up doses of pyriproxyfen sufficient to sterilize them. The possibilities of cross resistance to pyriproxyfen in this strongly pyrethroid resistant *Cx. quinquefasciatus* population also cannot be ruled out. Further studies need to be performed to investigate these hypotheses under controlled laboratory conditions.

Notwithstanding the lack of sterilization, Olyset Duo provided better personal protection against *Cx. quinquefasciatus* than Olyset Net (53% vs. 92%). This suggests that even though a significant reduction in the abundance of *Cx. quinquefasciatus* might not be expected from community-wide use of Olyset Duo, the mixture LN may still provide better protection against this species than the pyrethroid-only LN. While the impact on malaria vectors is of primary interest, the capacity of Olyset Duo to improve personal protection against *Cx. quinquefasciatus*, may improve acceptability to LN users [30].

## Conclusion

By killing more pyrethroid resistant *An. gambiae* and sterilizing surviving blood-fed females through the pyriproxyfen component, Olyset Duo has potential to provide better control of malaria transmission than pyrethroid only LNs in areas where pyrethroid resistance is compromising the efficacy of current LNs. The apparent lack of impact of pyriproxyfen on *Culex quinquefasciatus* mosquitoes requires further investigation. A community randomised trial is necessary to demonstrate whether the sterilizing effect of Olyset Duo will provide additional malaria transmission control over Olyset Net.
